# The Kotake–Narasimhan theorem in general ultradifferentiable classes

**DOI:** 10.1007/s13398-024-01586-z

**Published:** 2024-04-09

**Authors:** Stefan Fürdös

**Affiliations:** 1 Instituto de Matemática e Estatística, Universidade de São Paulo, Rua do Matão 1010, 05508-090 São Paulo, SP Brazil; 2 Fakultät für Mathematik, Universität Wien, Oskar-Morgenstern-Platz 1, 1090 Wien, Austria

**Keywords:** Kotake–Narasimhan theorem, Problem of iterates, Ultradifferentiable functions, Ultradifferentiable vectors, Primary 35B65, Secondary 26E10, 35B45, 35J48, 46E10

## Abstract

We prove a Kotake–Narasimhan type theorem in general ultradifferentiable classes given by weight matrices. In doing so we simultaneously recover and generalize significantly the known results for classes given by weight sequences and weight functions. In particular, we obtain a sharp Kotake–Narasimhan theorem for Beurling classes.

## Introduction

### Historical background

In this paper we consider the problem of iterates for elliptic operators with coefficients in general ultradifferentiable structures.^1^ In its general form the problem of iterates for an ultradifferentiable structure $\mathcal {U}$ can be stated in the following way:

Let *u* be a smooth function which satisfies the defining estimates of $\mathcal {U}$ with respect to the iterates of a differential operator *P*. Can we conclude that *u* is already an element of $\mathcal {U}$?

If the answer to the question above is affirmative for an ultradifferentiable structure $\mathcal {U}$ and an operator *P* then we say that the theorem of iterates holds for the operator *P* with respect to $\mathcal {U}$.

Recently, there has been a resurged interest in the problem of iterates in various different settings, see e.g. [6, 14, 17, 20, 21]. For surveys on the problem of iterates we refer the reader to [9, 16]. In this paper we are going to revisit the classical case of elliptic operators in open sets of ${\mathbb {R}}^n$ in view of the recently expanded theory on general ultradifferentiable classes.

The main starting point of the problem of iterates was when in 1962 Kotake–Narasimhan [27] and Komatsu [24] separately proved the following statement: If *P* is an elliptic operator of order *d* with analytic coefficients on an open set $\Omega \subseteq {\mathbb {R}}^n$, then a smooth function $u\in \mathcal {E}(\Omega )$ is analytic in $\Omega$ if and only if for each relatively compact set $U\subseteq \Omega$ there are constants $C,h>0$ such that

$$\begin{aligned} \Vert {P^ku} \Vert _{L^2(U)}\le Ch^k(dk)!, \quad k\in \mathbb {N}_0. \end{aligned}$$

Nelson [33] proved an analogous statement for an elliptic system of analytic vector fields.

The next natural step is then to ask if a similar result holds if one considers instead of the analytic class more general ultradifferentiable classes. We are going to say that the Kotake–Narasimhan theorem holds for an ultradifferentiable structure $\mathcal {U}$ if the theorem of iterates with respect to $\mathcal {U}$ holds for every elliptic operator *P* with coefficients in $\mathcal {U}$.

For example Bolley-Camus [7] proved the Kotake–Narasimhan theorem for Gevrey classes. If we want to consider more general families of ultradifferentiable classes then the commonly used spaces are the Denjoy–Carleman classes which are defined by weight sequences, for an introduction see e.g. [25], and the Braun–Meise–Taylor classes which are given by weight functions, introduced in their modern form by [13]. Both of these classes are generalizations of the Gevrey classes, however, they do not in general coincide, see [11]. In this paper we take a step further and consider ultradifferentiable classes given by weight matrices, i.e. countable families of weight sequences, which were introduced in [34, 35]. These classes include both Denjoy–Carleman and Braun-Meise-Taylor classes. In [20] we showed that the theorem of iterates with respect to classes given by weight matrices holds for elliptic operators with analytic coefficients using a microlocal analytic approach, generalizing results of [8] in the case of weight sequences and of [5] for weight functions.

More generally, the Kotake–Narasimhan theorem for Denjoy–Carleman classes was proven in [29] and in a more general form in [15]. In the case of Braun–Meise–Taylor classes the Kotake–Narasimhan theorem was shown by [6]. All these instances followed generally the lines of the proofs of [24, 27] and [7], which used a technique involving a-priori $L^2$-estimates and nested neighborhoods first introduced by Morrey-Nirenberg [32]. In this paper we prove the Kotake–Narasimhan theorem for ultradifferentiable classes given by weight matrices by adapting the proof in [7] resp. [14]. In doing so we not only recover the known statements in Denjoy–Carleman classes and Braun-Meise-Taylor classes but also partially generalizing them. In particular, in the case of Denjoy–Carleman classes Lions and Magenes [29] asked, what the optimal conditions on the weight sequences are in order for the Kotake–Narasimhan Theorem to hold. As we will see, our main result implies especially that the Kotake–Narasimhan Theorem holds for the Denjoy–Carleman classes determined by the sequences

$$\begin{aligned} N_k^q=q^{k^2},\qquad k\in \mathbb {N}_0, \end{aligned}$$

where $q>1$ is a real parameter. These sequences have not been covered by the previous works on the Kotake–Narasimhan Theorem for Denjoy–Carleman classes, cf. Remark 1.5.

### Statement of main results

In order to formulate our main results for weight sequences and weight functions we need to fix some notations: $\Omega$ will always be an open set of ${\mathbb {R}}^n$ and we set $D_j=-i\partial _j=-i\partial _{x_j}$ where $\partial _{x_j}$ is the *j*-th partial derivative, $j=1,\dotsc ,n$. We denote the set of positive integers by $\mathbb {N}$ whereas $\mathbb {N}_0=\mathbb {N}\cup \{0\}$. Furthermore we say that a sequence $\textbf{M}=(M_k)_k$ of positive numbers is a *weight sequence* if $M_0=1\le M_1$ and

1.1 $$\begin{aligned} M_{k}^2\le M_{k-1}M_{k+1} \end{aligned}$$

for all $k\in \mathbb {N}$. Next we define the Denjoy–Carleman class associated to $\textbf{M}$ over an open set $\Omega \subseteq {\mathbb {R}}^n$. To a weight sequence $\textbf{M}$ we can in fact associate two different ultradifferentiable classes. First the Roumieu class associated to $\textbf{M}$ is given by

$$\begin{aligned} \mathcal {E}^{\{ \textbf{M} \}} \left( \Omega \right)&=\biggl \{f\in \mathcal {E}(\Omega )\;\Big \vert \;\,\forall \,U\Subset \Omega \;\,\exists \,h>0 \;\,\exists \,C>0:\\&\qquad \qquad \qquad \qquad \qquad \sup _{x\in U}\,|D^\alpha f(x)|\le Ch^{|\alpha |}M_{|\alpha |}\; \;\,\forall \,\alpha \in \mathbb {N}_0^n\biggr \} \end{aligned}$$

whereas the Beurling class associated to $\textbf{M}$ is

$$\begin{aligned} \mathcal {E}^{( \textbf{M} )} ( \Omega )&=\biggl \{f\in \mathcal {E}(\Omega )\;\Big \vert \;\,\forall \,U\Subset \Omega \;\,\forall \,h>0 \;\,\exists \,C>0:\\&\qquad \qquad \qquad \qquad \qquad \sup _{x\in U}\,|D^\alpha f(x)|\le Ch^{|\alpha |}M_{|\alpha |}\; \;\,\forall \,\alpha \in \mathbb {N}_0^n\biggr \}. \end{aligned}$$

A basic question in the theory of ultradifferentiable classes is that of quasianalyticity. We recall that an algebra *E* of smooth functions is called non-quasianalytic if the only flat function in *E* is the zero function. The Denjoy–Carleman theorem, see [23], says that a Denjoy–Carleman class $\mathcal {E}^{[ \textbf{M} ]} ( \Omega )$^2^ is non-quasianalytic if and only if

1.2 $$\begin{aligned} \sum _{k=1}^\infty \frac{M_{k-1}}{M_k}<\infty . \end{aligned}$$

We call a weight sequence $\textbf{M}$ non-quasianalytic if (1.2) holds and otherwise quasianalytic.

In order to formulate the Kotake–Narasimhan Theorem for Denjoy–Carleman classes we have to specify additional conditions on the weight sequence $\textbf{M}$. It is often easier to formulate these conditions not in terms of the sequence $(M_k)_k$ directly but to use other sequences associated to $\textbf{M}$, like $m_k=M_k/k!$ or $\mu _k=M_k/M_{k-1}$.

#### Definition 1.1

Let $\textbf{M}=(M_k)_k$ be a weight sequence. We say that $\textbf{M}$ is *weakly regular* if the following conditions hold:

1.3 $$\begin{aligned} \lim _{k\rightarrow \infty } \root k \of {m_k}=\infty ,\end{aligned}$$

1.4 $$\begin{aligned} \text {the sequence }\root k \of {m_k} \text { is increasing,}\end{aligned}$$

1.5 $$\begin{aligned} \;\,\exists \,\gamma >0:\; M_{k+1}\le \gamma ^{k+1}M_k\quad \;\,\forall \,k\in \mathbb {N}_0. \end{aligned}$$

#### Example 1.2

Let $s\ge 1$. The Gevrey sequence $\textbf{G}^{s}$, which is given by $G^s_k=(k!)^s$, is weakly regular. More generally the weight sequences $\textbf{B}^{s,\sigma }$, defined by $B^{s,\sigma }_k=(k!)^s\log (k+e)^{\sigma k}$, are weakly regular for all $s\ge 1$ and $\sigma >0$. The sequences $\textbf{B}^{s,\sigma }$ are quasianalytic if and only if $s=1$ and $0<\sigma \le 1$.

Another examples of weakly regular weight sequences are the following: For $q>1$ and $1<r$ let $\textbf{L}^{q,r}$ be given by $L_k^{q,r}=q^{k^r}$. The weight sequences $\textbf{L}^{q,r}$ are weakly regular for all $q>1$ and $1<r\le 2$. In particular the case $r=2$ are the *q*-Gevrey sequences $\textbf{N}^q$ given by $N^q=q^{k^2}$.

If $\textsf{P}=\{P_1,\dotsc ,P_\ell \}$ is a system of partial differential operators

$$\begin{aligned} P_j=\sum _{{|\alpha |}\le d_j} a_{j\alpha }D^\alpha \end{aligned}$$

with smooth coefficients $a_{j\alpha }\in \mathcal {E}(\Omega )$, then we recall that the system $\textsf{P}$ is *elliptic* in $\Omega$ if for every $x\in \Omega$ the principal symbols

$$\begin{aligned} p_j(x,\xi )=\sum _{{|\alpha |}=d_j} a_{j\alpha }(x)\xi ^\alpha , \qquad \xi \in {\mathbb {R}}^n, \end{aligned}$$

have no common nontrivial real zero in $\xi$.

Our main result in the case of Denjoy–Carleman classes is the following theorem.

#### Theorem 1.3

Let $\textbf{M}$ be a weakly regular weight sequence and $\textsf{P}=\{P_1,\dotsc ,P_\ell \}$ be an elliptic system of differential operators. Then the following statements hold:

1. Assume that the coefficients of the operators $P_j$, $j=1,\dotsc ,\ell$, are all elements of $\mathcal {E}^{\{ \textbf{M} \}} \left( \Omega \right)$. Then $u\in \mathcal {E}^{\{ \textbf{M} \}} \left( \Omega \right)$ if and only if $u\in \mathcal {E}(\Omega )$ and for all $U\Subset \Omega$ there are constants $C,h>0$ such that for all $k\in \mathbb {N}_0$ we have that
  $$\begin{aligned} \Vert {P^\alpha u} \Vert _{L^2(U)}\le Ch^{d_\alpha }M_{d_\alpha } \end{aligned}$$
  for all $\alpha \in \{1,\dotsc ,\ell \}^k$ where $P^{\alpha }=P_{\alpha _1}\dots P_{\alpha _k}$ and $d_\alpha =d_{\alpha _1}+\dots +d_{\alpha _k}$ with $d_j$ denoting the order of the operator $P_j$, $j=1,\dotsc ,\ell$.
2. Assume that the coefficients of the operators $P_j$, $j=1,\dotsc ,\ell$, are in $\mathcal {E}^{( \textbf{M} )} ( \Omega )$. Then $u\in \mathcal {E}^{( \textbf{M} )} ( \Omega )$ if and only if $u\in \mathcal {E}(\Omega )$ and for all $U\Subset \Omega$ and every $h>0$ there exists a constant $C>0$ such that
  $$\begin{aligned} \Vert {P^\alpha u} \Vert _{L^2(U)}\le Ch^{d_\alpha }M_{d_\alpha } \end{aligned}$$
  for all *k* and $\alpha \in \{1,\dotsc ,\ell \}^k$.

#### Remark 1.4

The notion of a weakly regular weight sequence is inspired by [18]. In that article a weight sequence $\textbf{M}$ is called regular if $\textbf{M}$ satisfies (1.3), (1.5) and instead of (1.4) the sequence $\textbf{M}$ is strongly logarithmic convex, i.e.

1.6 $$\begin{aligned} m_{k}^2\le m_{k-1}m_{k+1}\qquad \;\,\forall \,k\in \mathbb {N}. \end{aligned}$$

In fact, (1.6) implies (1.4).

#### Remark 1.5

We need to point out that Theorem 1.3 is a considerably more general statement then the previous known results, like [15, 29] or [14] in the Roumieu case and [12] in the Beurling case. In these papers varying conditions on the weight sequence are assumed, but these conditions always include (1.2),

1.7 $$\begin{aligned} m_{k}m_\ell \le m_{k+\ell }\qquad \;\,\forall \,k,\ell \in \mathbb {N}_0 \end{aligned}$$

and

1.8 $$\begin{aligned} \;\,\exists \,\gamma >0:\; M_{j+k}\le \gamma ^{j+k+1} M_{j}M_k,\quad \;\,\forall \,j,k\in \mathbb {N}_0. \end{aligned}$$

To compare these conditions with those of our result we may note that it is easy to see that (1.7) is another consequence of (1.6). However, if $\textbf{M}$ is a weight sequence we can see that (1.4) implies also (1.7): For $k=0$ we have that $m_0m_\ell =m_\ell$ for all $\ell \in \mathbb {N}_0$ and the same is true for $\ell =0$ and all $k\in \mathbb {N}_0$. So we have to show (1.7) for $k,\ell \in \mathbb {N}$, but then we have

$$\begin{aligned} m_km_\ell \le m_{k+\ell }^{k/(k+\ell )}m_{k+\ell }^{\ell /(k+\ell )}=m_{k+\ell } \end{aligned}$$

by (1.4). Thence in that regard our conditions are formally more restrictive, but this is compensated by the fact that we replaced the other conditions noted above by far weaker conditions.

The most interesting property above in that regard is the last one. It is clear that (1.8) implies (1.5). However, (1.8) is far more restrictive than (1.5), see e.g. [30]. In fact, if a weight sequence $\textbf{M}$ satisfies (1.8) then there is some $s>1$ such that $\mathcal {E}^{[ \textbf{M} ]} ( \Omega )\subseteq \mathcal {G}^s(\Omega )$. For example, the sequences $\textbf{L}^{q,r}$ cannot satisfy (1.8) for any choice of $q,r>1$.

Moreover, we have replaced (1.2) by the non-analyticity condition (1.3). We will see in the next section, cf. Remark 2.4, that (1.3) is still nearly superfluous but we use it in order to allow for a unified formulation of Theorem 1.3. This only excludes formally the analytic case, which is well-known to hold.

We may also observe that in the Beurling case Theorem 1.3 is also a strict statement in the sense that we allow that the coefficients are in the class given by the same weight sequence as the space of vectors considered. In contrast, for example [12] requires that the coefficients of the operators are in a strictly smaller class than the vectors considered. We are able to remove this restriction by applying an argument given in [26], which essentially allows us to reduce the Beurling case to the Roumieu case.

In the case of Braun–Meise–Taylor classes our main theorem boils down to the following statement. Recall that a weight function $\omega$ is an increasing continuous function $\omega : [0,\infty )\rightarrow [0,\infty )$ satisfying $\omega (t)=0$ for $t\in [0,1]$ and 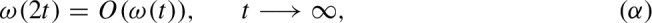

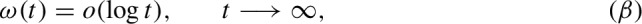

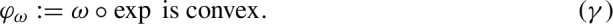
 The conjugate function $$\varphi ^*_\omega (t) =\sup _{s\ge 0} (st-\varphi _\omega (s))$$, $$t\ge 0$$, is also convex, continuous and increasing and $$\varphi ^{**}_\omega =\varphi _\omega $$. A smooth function $$f\in \mathcal {E}(\Omega )$$ is an element of the Roumieu class $$\mathcal {E}^{\{ \omega \}} \left( \Omega \right) $$ (resp. the Beurling class $$\mathcal {E}^{( \omega )} ( \Omega )$$) if for any $$U\Subset \Omega $$ there are constants $$C,h>0$$ (resp. for all $$h>0$$ there is a constant $$C=C_{h,U}$$) such that

$$\begin{aligned} \sup _{x\in U}|D^\alpha f(x)|\le Ce^{\tfrac{1}{h}\varphi _\omega ^*(h{|\alpha |})}\qquad \;\,\forall \,\alpha \in \mathbb {N}_0^n. \end{aligned}$$

#### Theorem 1.6

Let $\omega$ be a concave weight function such that $\omega (t)=o(t)$ and $\textsf{P}=\{P_1,\dotsc ,P_\ell \}$ be an elliptic system of differential operators.

1. Assume that all coefficients of the operators $P_j$, $j=1,\dotsc ,\ell$, are in $\mathcal {E}^{\{ \omega \}} \left( \Omega \right)$. Then $u\in \mathcal {E}^{\{ \omega \}} \left( \Omega \right)$ if and only if $u\in \mathcal {E}(\Omega )$ and for all $U\Subset \Omega$ there are constants $C,h>0$ such that
  $$\begin{aligned} \Vert {P^\alpha u} \Vert _{L^2(U)}\le Ce^{\tfrac{1}{h}\varphi _\omega ^*(hd_\alpha )} \end{aligned}$$
  for all $\alpha \in \{1,\dotsc ,\ell \}^k$ and every $k\in \mathbb {N}_0$.
2. Assume that all coefficients of the $P_j$, $j=1,\dotsc ,\ell$ are in $\mathcal {E}^{( \omega )} ( \Omega )$. Then $u\in \mathcal {E}^{( \omega )} ( \Omega )$ if and only if $u\in \mathcal {E}(\Omega )$ and for all $U\Subset \Omega$ and every $h>0$ there is a constant $C>0$ such that
  $$\begin{aligned} \Vert {P^\alpha u} \Vert _{L^2(U)}\le Ce^{\tfrac{1}{h}\varphi _\omega ^*(hd_\alpha )} \end{aligned}$$
  for all $\alpha \in \{1,\dotsc ,\ell \}^k$ and every $k\in \mathbb {N}_0$.

#### Remark 1.7

Theorem 1.6 generalizes the main theorem in [6] from a single elliptic operator to elliptic systems of operators. Furthermore, in the Beurling case the Kotake–Narasimhan theorem was proven in [6], again, only for operators whose coefficients are in a strictly smaller class $\mathcal {E}^{( \tau )} ( \Omega )\subsetneq \mathcal {E}^{( \omega )} ( \Omega )$.

As we have announced above we are going to prove our main Theorem 2.14 in the far more general setting of weight matrices, that is countable families of weight sequences. We utilize the approach of [7], which in particular allows us to apply the aforementioned technique of [26] in order to prove the Beurling version of Theorem 2.14 by effectively reducing it to the Roumieu case even in the case of weight matrices. However, we need to point out that in general it is not possible to use this technique in the case of non-trivial weight matrices, see for example [19, Section 7].

The structure of the paper is the following: In Sect. 2 we recall basic definitions and facts about weight sequences, weight matrices and the ultradifferentiable classes of functions and vectors generated by them. Having sufficiently developed the theory of ultradifferentiable structures given by weight matrices we can formulate our main result Theorem 2.14 at the end of Sect. 2. In Sect. 3 we prove the fundamental $L^2$-estimate which is used in Sect. 4 to prove Theorem 2.14. We conclude the paper by stating some remarks in Sect. 5.

## Ultradifferentiable structures

### Weight sequences

The following properties of a weight sequence are well known.

#### Lemma 2.1

Let $\textbf{M}$ be a weight sequence and set $\Lambda _k=\root k \of {M_k}$ and $\mu _k=\frac{M_k}{M_{k-1}}$ for $k\in \mathbb {N}$. Then the following holds:

1. $$M_jM_k\le M_{j+k}$$ for all $$j,k\in \mathbb {N}_0$$.
2. The sequence $\Lambda _k$ is increasing, i.e. $\Lambda _k\le \Lambda _{k+1}$ for all $k\in \mathbb {N}$.
3. The sequence $\mu _k$ is increasing.
4. $$\Lambda _k\le \mu _k$$ for all *k*.

For later convenience we shall take a closer look at the structure of Denjoy–Carleman classes, for more details see [25]. Here we do not to require that $\textbf{M}$ is a weight sequence.

So let $U\subseteq {\mathbb {R}}^n$ be an open set, $\textbf{M}$ be an increasing sequence of positive numbers and $h>0$ be a parameter. Then $\mathcal {E}(\overline{U})$ is the space of smooth functions *f* on *U* such that $\partial ^\alpha f$ extends continuously to $\overline{U}$ and we define a seminorm on $\mathcal {E}(\overline{U})$ by setting

$$\begin{aligned} \Vert {f} \Vert _{\textbf{M},U,h}:=\sup _{\begin{array}{c} x\in U\\ \alpha \in \mathbb {N}_0^n \end{array}}\frac{|D^\alpha f(x)|}{h^{|\alpha |}M_{|\alpha |}}. \end{aligned}$$

Thusis a Banach space. Then $\mathcal {B}^{\{ \textbf{M} \}} ( U )=\mathop {\textrm{ind}}\limits _{h>0}\mathcal {B}_{ \textbf{M} , h }(U)=\bigcup _{h>0} \mathcal {B}_{ \textbf{M} , h }(U)$ resp. $\mathcal {B}^{( \textbf{M} )} ( U )=\mathop {\textrm{proj}}\limits _{h>0}\mathcal {B}_{ \textbf{M} , h }(U)=\bigcap _{h>0}\mathcal {B}_{ \textbf{M} , h }(U)$ is the Roumieu resp. Beurling class of global ultradifferentiable functions associated to the weight sequence $\textbf{M}$ over *U*.

The local ultradifferentiable classes $\mathcal {E}^{[ \textbf{M} ]} ( U )$ associated to $\textbf{M}$ over *U* can thus be described as $\mathcal {E}^{[ \textbf{M} ]} ( U )=\mathop {\textrm{proj}}\limits _{V\Subset U}\mathcal {B}^{[ \textbf{M} ]} ( V )$. If $\textbf{M}$ and $\textbf{N}$ are two sequences we write

$$\begin{aligned} \textbf{M}\le \textbf{N}\quad&:\Longleftrightarrow \quad \;\,\forall \,k\in \mathbb {N}_0: M_k\le N_k, \\ \textbf{M}\preceq \textbf{N}\quad&:\Longleftrightarrow \quad \left( \tfrac{M_k}{N_k}\right) ^{1/k} \text { is bounded for } k\rightarrow \infty , \\ \textbf{M}\lhd \textbf{N}\quad&:\Longleftrightarrow \quad \left( \tfrac{M_k}{N_k}\right) ^{1/k}\longrightarrow 0 \text { if } k\rightarrow \infty . \end{aligned}$$

If $\textbf{M}\preceq \textbf{N}$ then $\mathcal {B}^{[ \textbf{M} ]} ( U )\subseteq \mathcal {B}^{[ \textbf{N} ]} ( U )$ and $\mathcal {E}^{[ \textbf{M} ]} ( U )\subseteq \mathcal {E}^{[ \textbf{N} ]} ( U )$. We are going to write $\textbf{M}\approx \textbf{N}$ if $\textbf{M}\preceq \textbf{N}$ and $\textbf{N}\preceq \textbf{M}$. Furthermore $\textbf{M}\lhd \textbf{N}$ implies $\mathcal {B}^{\{ \textbf{M} \}} ( U )\subseteq \mathcal {B}^{( \textbf{N} )} ( U )$ and $\mathcal {E}^{\{ \textbf{M} \}} \left( U \right) \subseteq \mathcal {E}^{( \textbf{N} )} ( U )$.

We recall that to a weight sequence $\textbf{M}$ (or an abritary sequence) we associate another sequence $m_k=M_k/k!$. It follows from above that the condition

2.1 $$\begin{aligned} \lim _{k\rightarrow \infty }\root k \of {m_k}=\infty \end{aligned}$$

implies that $\mathcal {A}(U)\subsetneq \mathcal {E}^{[ \textbf{M} ]} ( U )$. We are also discussing the other conditions appearing in the beginning of this article, e.g.

2.2 $$\begin{aligned} m_k^2\le m_{k-1}m_{k+1},\qquad \;\,\forall \,k\in \mathbb {N},\end{aligned}$$

2.3 $$\begin{aligned} \;\,\exists \,C>0:\; M_{k+1}\le C^{k+1}M_k\quad \;\,\forall \,k\in \mathbb {N}_0, \end{aligned}$$

#### Remark 2.2

1. If $\textbf{M}$ is a weight sequence satisfying (2.1) and (2.2) then clearly $\textbf{m}=(m_k)_k$ is also a weight sequence. In particular
  2.4 $$\begin{aligned} m_{j}m_k\le m_{j+k}\qquad j,k\in \mathbb {N}_0. \end{aligned}$$
  Furthermore, we have the following property:
  2.5 $$\begin{aligned} \text {The sequence }\root k \of {m_k}=\frac{\Lambda _k}{k!^{1/k}} \text { is increasing.} \end{aligned}$$
  Therefore the sequence $\Lambda _k$ is strictly increasing.
2. The estimate (2.3) is equivalent to
  $$\begin{aligned} \;\,\exists \,C>0:\; m_{k+1}\le C^{k+1}m_k\qquad \;\,\forall \,k\in \mathbb {N}_0. \end{aligned}$$

For later use we note the following Lemma.

#### Lemma 2.3

Let $\textbf{M}$ be a sequence with $M_0=1\le M_1$ satisfying (2.1). Then the following statements hold:

1. If (2.2) is satisfied then $\mu _k+1\le \mu _{k+1}$ for all $k\in \mathbb {N}$.
2. Assume that the sequence $\root k \of {m_k}$ is increasing, i.e. (2.5) holds. If we consider the sequence $\Theta _k=k\root k \of {m_k}$ then we have also that
  2.6 $$\begin{aligned} \Theta _k+1\le \Theta _{k+1}\qquad \;\,\forall \,k\in \mathbb {N}. \end{aligned}$$

#### Proof

Recall from Remark 2.2 that $\textbf{m}$ is a weight sequence and hence we can apply Lemma 2.1. By Lemma 2.1(3) we thus have that the sequence $\mu _k/k$ is increasing and therefore

$$\begin{aligned} \mu _k+\frac{\mu _k}{k}\le \mu _{k+1}\qquad \;\,\forall \,k\in \mathbb {N}. \end{aligned}$$

This gives instantly (1) since $\mu _k/k\ge 1$.

For (2) we need only to observe that

$$\begin{aligned} k\root k \of {m_k}+1\le k\root k+1 \of {m_{k+1}}+1\le (k+1)\root k+1 \of {m_{k+1}} \end{aligned}$$

for all $k\in \mathbb {N}$ since $\root k \of {m_k}\ge 1$. $\square$

#### Remark 2.4

We recall that condition (2.1) implies that $\mathcal {A}(U)\subsetneq \mathcal {E}^{[ \textbf{M} ]} ( U )$. However, it is nearly superfluous in view of (2.2): Iterating the estimate in Lemma 2.3(1) we obtain that for any strongly logarithmically convex weight sequence $\textbf{M}$ we have that $k\le \mu _k$ and therefore $k!\le M_k$. In particular $(k!)\preceq M_k$ which in turn implies that $\mathcal {A}(U)\subseteq \mathcal {E}^{\{ \textbf{M} \}} \left( U \right)$. In fact, for that relation to hold it is enough to assume that $\root k \of {m_k}$ is increasing, since this gives also that $\liminf _{k\rightarrow \infty }\root k \of {m_k}>0$ (recall that $m_1\ge 1$ by assumption).

In the Beurling situation, we know that (2.1) holds if and only if $\mathcal {A}(\mathbb {R})\subseteq \mathcal {E}^{( \textbf{M} )} ( \mathbb {R} )$. Thus we only formally exclude the analytic case by our assumptions, which of course has been well investigated.

In order to deal later with the Beurling case we need the following statement, which will allow us to reduce the key argument in the Beurling case to the Roumieu case. Its proof is based on the proof of [26, Lemma 6], cf. also [20, Lemma 2.2].

#### Proposition 2.5

Let $\textbf{M}$ be a weight sequence satisfying (2.1) and (2.5). If $\textbf{L}=(L_k)_k$ is a sequence of positive numbers such that $\textbf{L}\lhd \textbf{M}$ then there is a sequence $\textbf{N}$ with $N_0=1\le N_1$ satisfying (2.1) and (2.5) such that

$$\begin{aligned} \textbf{L}\le \textbf{N}\lhd \textbf{M}. \end{aligned}$$

#### Proof

We set $m_k=M_k/k!$ and $\ell _k=L_k/k!$. Then the condition $\textbf{L}\lhd \textbf{M}$ implies that for all $h>0$ there is a constant $C_h$ such that

2.7 $$\begin{aligned} \ell _k\le C_h h^km_k\qquad \;\,\forall \,k\in \mathbb {N}_0. \end{aligned}$$

For $h>0$ we take $C_h^\bullet$ to be the smallest number such that (2.7) holds. We set

2.8 $$\begin{aligned} {\tilde{\ell }}_k=\inf _{h>0} C^\bullet _hh^km_k,\qquad k\in \mathbb {N}_0. \end{aligned}$$

and define a auxillary sequence by ${\bar{\ell }}_k= {\tilde{\ell }}_k/{\tilde{\ell }}_0$. Since the infimum in (2.8) is assumed for some $h>0$ we have that

$$\begin{aligned} \left( \frac{m_k}{{\bar{\ell }}_k}\right) ^2=\frac{{\tilde{\ell }}_0}{C_h^\bullet h^{k-1}}\frac{{\tilde{\ell }}_0}{C_h^\bullet h^{k+1}} \le \frac{m_{k-1}}{{\bar{\ell }}_{k-1}}\frac{m_{k+1}}{{\bar{\ell }}_{k+1}},\qquad k\in \mathbb {N}. \end{aligned}$$

Hence the sequence $(m_k/{\bar{\ell }}_k)_k$ is logarithmic convex and by definition ${\bar{\ell }}_0= 1$ and therefore the sequence

$$\begin{aligned} c_k=\frac{\root k \of {m_k}}{\root k \of {{\bar{\ell }}_k}} \end{aligned}$$

is increasing since Lemma 2.1(2) still holds in that situation. Furthermore $c_k\rightarrow \infty$ because $\textbf{L}\lhd \textbf{M}$.

Now we define the sequence $\textbf{N}=(N_k)_k$ by $N_0=1$ and $N_k=k!n_k$ for $k\in \mathbb {N}$, where

$$\begin{aligned} \root k \of {n_k}=\max \left\{ \root 2k \of {m_k};\max _{j\le k}\frac{\root j \of {m_j}}{c_j}\right\} . \end{aligned}$$

Clearly the sequence $\root k \of {n_k}$ is increasing. Moreover, $\root k \of {n_k}\ge \root 2k \of {m_k}\rightarrow \infty$ for $k\rightarrow \infty$. Thence the sequence $\textbf{N}$ satisfies the conditions (2.1) and (2.5).

Finally we observe that $L_k\le k!{\bar{\ell }}_k\le k! n_k=N_k$ and

$$\begin{aligned} \root k \of {\frac{n_k}{m_k}}=\max \left\{ \frac{\root 2k \of {m_k}}{\root k \of {m_k}}; \max _{{j\le k}}\frac{\root j \of {m_j}}{c_j\root k \of {m_k}}\right\} \longrightarrow 0. \end{aligned}$$

Thence $$\textbf{L}\le \textbf{N}\lhd \textbf{M}$$. $$\square $$

### Weight matrices

Now we are in the position to introduce the concept of weight matrices.

#### Definition 2.6

A weight matrix $\mathfrak {M}$ is a family of weight sequences such that for all $\textbf{M},\textbf{N}\in \mathfrak {M}$ we have either $\textbf{M}\le \textbf{N}$ or $\textbf{N}\le \textbf{M}$.

If $\mathfrak {M}$ is a weight matrix then the Roumieu class of global ultradifferentiable functions associated to $\mathfrak {M}$ is

$$\begin{aligned} \mathcal {B}^{\{ \mathfrak {M} \}} ( U )&=\mathop {\textrm{ind}}\limits _{\begin{array}{c} \textbf{M}\in \mathfrak {M}\\ h>0 \end{array}}\mathcal {B}_{ \textbf{M} , h }(U) \end{aligned}$$

whereas the Beurling class is

$$\begin{aligned} \mathcal {B}^{( \mathfrak {M} )} ( U )&=\mathop {\textrm{proj}}\limits _{\begin{array}{c} \textbf{M}\in \mathfrak {M}\\ h>0 \end{array}}\mathcal {B}_{ \textbf{M} , h }(U). \end{aligned}$$

Then the local ultradifferentiable classes associated to $\mathfrak {M}$ are given by

$$\begin{aligned} \mathcal {E}^{[ \mathfrak {M} ]} ( U )&=\mathop {\textrm{proj}}\limits _{V\Subset U}\mathcal {B}^{[ \mathfrak {M} ]} ( V ). \end{aligned}$$

If $\mathfrak {M}$ and $\mathfrak {N}$ are two weight matrices then we set

$$\begin{aligned} \mathfrak {M}&\{\preceq \}\mathfrak {N}{} & {} \Longleftrightarrow&\;\,\forall \,\textbf{M}\in \mathfrak {M}\;\;\,\exists \,\textbf{N}\in \mathfrak {M}:\;\; \textbf{M}&\preceq \textbf{N},\\ \mathfrak {M}&(\preceq )\mathfrak {N}{} & {} \Longleftrightarrow&\;\,\forall \,\textbf{N}\in \mathfrak {N}\; \;\,\exists \,\textbf{M}\in \mathfrak {M}:\;\;\textbf{M}&\preceq \textbf{N},\\ \mathfrak {M}&(\lhd \}\mathfrak {N}{} & {} \Longleftrightarrow&\;\,\forall \,\textbf{M}\in \mathfrak {M}\;\;\,\forall \,\textbf{N}\in \mathfrak {N}:\;\;\textbf{M}&\lhd \textbf{N}. \end{aligned}$$

We write $[\preceq ]=\{\preceq \},(\preceq )$. Clearly $\mathcal {B}^{[ \mathfrak {M} ]} ( U )\subseteq \mathcal {B}^{[ \mathfrak {N} ]} ( U )$, $\mathcal {E}^{[ \mathfrak {M} ]} ( \Omega )\subseteq \mathcal {E}^{[ \mathfrak {N} ]} ( \Omega )$ if $\mathfrak {M}[\preceq ]\mathfrak {N}$ and $\mathcal {B}^{\{ \mathfrak {M} \}} ( U )\subseteq \mathcal {B}^{( \mathfrak {N} )} ( U )$, $\mathcal {E}^{\{ \mathfrak {M} \}} \left( \Omega \right) \subseteq \mathcal {E}^{( \mathfrak {N} )} ( \Omega )$ when $\mathfrak {M}(\lhd \} \mathfrak {N}$. We set also $\mathfrak {M}[\approx ]\mathfrak {N}$ if $\mathfrak {M}[\preceq ]\mathfrak {N}$ and $\mathfrak {N}[\preceq ]\mathfrak {M}$. Then $\mathcal {B}^{[ \mathfrak {M} ]} ( U )=\mathcal {B}^{[ \mathfrak {N} ]} ( U )$, $\mathcal {E}^{[ \mathfrak {M} ]} ( \Omega )=\mathcal {E}^{[ \mathfrak {N} ]} ( \Omega )$ for $\mathfrak {M}[\approx ]\mathfrak {N}$.

#### Definition 2.7

Let $\mathfrak {M}$ be a weight matrix.

1. We say that $\mathfrak {M}$ is *R*-semiregular if the following conditions hold:
  2.9 $$\begin{aligned}{} & {} \;\,\forall \,\textbf{M}\in \mathfrak {M}:\quad \lim _{k\rightarrow \infty }\root k \of {m_k}=\infty ,\end{aligned}$$
  2.10 $$\begin{aligned}{} & {} \;\,\forall \,\textbf{M}\in \mathfrak {M}\;\,\exists \,\textbf{N}\in \mathfrak {M}\;\,\exists \,q>0:\;\, M_{k+1}\le q^{k+1}N_k,\qquad k\in \mathbb {N}_0. \end{aligned}$$
2. $\mathfrak {M}$ is *B*-semiregular if (2.9) and
  2.11 $$\begin{aligned} \;\,\forall \,\textbf{N}\in \mathfrak {M}\;\,\exists \,\textbf{M}\in \mathfrak {M}\;\,\exists \,q>0:\;\, M_{k+1}\le q^{k+1}N_k,\qquad k\in \mathbb {N}_0, \end{aligned}$$
  are satisfied.

We write [semiregular$]=R$-semiregular, *B*-semiregular.

#### Remark 2.8

Let $d\in \mathbb {N}$ be fixed and $\mathfrak {M}$ be a weight matrix.

1. If $\mathfrak {M}$ is *R*-semiregular then the following holds:
  2.12 $$\begin{aligned} \;\,\forall \,\textbf{M}\in \mathfrak {M}\; \;\,\exists \,\textbf{N}\in \mathfrak {M}\; \;\,\exists \,q>0:\quad M_{k+d}\le q^{k+1}N_k,\qquad \;\,\forall \,k\in \mathbb {N}_0. \end{aligned}$$
2. If $\mathfrak {M}$ is *B*-semiregular then we have that:
  2.13 $$\begin{aligned} \;\,\forall \,\textbf{N}\in \mathfrak {M}\; \;\,\exists \,\textbf{M}\in \mathfrak {M}\; \;\,\exists \,q>0:\quad M_{k+d}\le q^{k+1}N_k,\qquad \;\,\forall \,k\in \mathbb {N}_0. \end{aligned}$$

The statements follow by iterating (2.10) and (2.11), respectively.

Let *U* be an open set in ${\mathbb {R}}^n$. We say that *U* has Lipschitz boundary if for all $x_0\in \partial U$ there are some $r>0$, local coordinates $(x_1,\dotsc ,x_n)$ and a Lipschitz function $h=h(x_1,\dotsc ,x_{n-1})$ such thatwhere $B(x_0,r)$ is the ball of radius *r* in ${\mathbb {R}}^n$ centered at $x_0$.

#### Remark 2.9

For completeness we give an alternative characterization of $\mathcal {E}^{[ \mathfrak {M} ]} ( \Omega )$ when $\mathfrak {M}$ is a [semiregular] weight matrix.

1. Let $U\subseteq {\mathbb {R}}^n$ be a bounded open set with Lipschitzian boundary. The Sobolev Theorem [1] implies that a smooth function $f\in \mathcal {E}(U)$ is an element of $\mathcal {B}^{\{ \mathfrak {M} \}} ( U )$ (of $\mathcal {B}^{( \mathfrak {M} )} ( U )$) if and only if there are constants $C,h>0$ and some $\textbf{M}\in \mathfrak {M}$ (for every $h>0$ and $\textbf{M}\in \mathfrak {M}$ there is a constant $C>0$) such that
  2.14 $$\begin{aligned} \Vert {D^\alpha f} \Vert _{L^2(U)}\le Ch^{|\alpha |}M_{|\alpha |},\qquad \;\,\forall \,\alpha \in \mathbb {N}_0^n. \end{aligned}$$
  We consider the more difficult Beurling case and leave the Roumieu case to the reader. Hence let $f\in \mathcal {E}(U)$ and suppose that for all $\textbf{M}\in \mathfrak {M}$ and $h>0$ there is some $C>0$ such that (2.14) is satisfied for all $\alpha \in \mathbb {N}_0^n$. By the Sobolev Imbedding Theorem we have that for integers $\sigma >n/2$ the following estimate holds
  2.15 $$\begin{aligned} \sup _{x\in U}|u(x)|\le A\Vert {u} \Vert _{H^\sigma (U)} \end{aligned}$$
  for all $u\in \mathcal {E}(U)$ where the constant *A* depends only on *U*, *n* and $\sigma$. We fix now $\sigma >n/2$. If we set $u= D^\alpha f$ then we obtain
  $$\begin{aligned} \begin{aligned} \Vert {D^\alpha f} \Vert _{H^\sigma (U)}^2&=\sum _{{|\beta |}\le \sigma }\Vert {D^{\alpha +\beta }f} \Vert _{L^2(U)}^2\\&\le C^2 \sum _{{|\beta |}\le \sigma }h^{2({|\alpha |}+{|\beta |})}M_{{|\alpha |}+{|\beta |}}^2\\&\le C^2 h^{2{|\alpha |}}M_{{|\alpha |}+\sigma }^2\sum _{{|\beta |}\le \sigma }h^{|\beta |}. \end{aligned} \end{aligned}$$
  Thence, using (2.13) we conclude that for all $\textbf{M}\in \mathfrak {M}$ and $h>0$ there is a constant $C>0$ such that
  $$\begin{aligned} \Vert {D^\alpha f} \Vert _{H^\sigma (U)}\le Ch^{|\alpha |}M_{|\alpha |},\qquad {|\alpha |}\in \mathbb {N}_0^n. \end{aligned}$$
  Thus applying also (2.15) gives that $f\in \mathcal {B}^{( \mathfrak {M} )} ( U )$. The other direction follows from the trivial estimate
  $$\begin{aligned} \Vert {g} \Vert _{L^2(U)}\le |U|\sup _{x\in U}|g(x)|,\qquad \;\,\forall \,g\in \mathcal {C}(U). \end{aligned}$$
2. On the other hand, it is easy to see that, if $\Omega \subseteq {\mathbb {R}}^n$ is an arbitrary open set and $f\in \mathcal {E}(\Omega )$ then $f\in \mathcal {E}^{[ \mathfrak {M} ]} ( \Omega )$ if and only if for all $x\in \Omega$ there is a neighborhood *U* of *x* such that $f\vert _U\in \mathcal {B}^{[ \mathfrak {M} ]} ( U )$.
3. Combining these two statements we have the following characterizations: Let $f\in \mathcal {E}(\Omega )$.
  If $\mathfrak {M}$ is an *R*-semiregular weight matrix then $f\in \mathcal {E}^{\{ \mathfrak {M} \}} \left( \Omega \right)$ if and only if for all $x\in \Omega$ there is a neighborhood *U* of *x* such that
    $$\begin{aligned} \;\,\exists \,\textbf{M}\in \mathfrak {M}\, \;\,\exists \,h>0\,\;\,\exists \,C>0:\quad \Vert {D^\alpha f} \Vert _{L^2(U)}\le Ch^{|\alpha |}M_{|\alpha |}\qquad \;\,\forall \,\alpha \in \mathbb {N}_0^n. \end{aligned}$$
  If $\mathfrak {M}$ is a *B*-semiregular weight matrix then $f\in \mathcal {E}^{( \mathfrak {M} )} ( \Omega )$ if and only if for all $x\in \Omega$ there is a neighborhood *U* of *x* such that
    $$\begin{aligned} \;\,\forall \,\textbf{M}\in \mathfrak {M}\,\;\,\forall \,h>0\,\;\,\exists \,C>0:\quad \Vert {D^\alpha f} \Vert _{L^2(U)}\le Ch^{|\alpha |}M_{|\alpha |}\qquad \;\,\forall \,\alpha \in \mathbb {N}_0^n. \end{aligned}$$

#### Definition 2.10

Let $\mathfrak {M}$ be a weight matrix.

1. We say that $\mathfrak {M}$ is weakly *R*-regular if (2.9), (2.10) and
  2.16 $$\begin{aligned} \;\,\forall \,\textbf{M}\in \mathfrak {M}:\quad \text {The sequence }\root k \of {m_k}\text { is increasing.} \end{aligned}$$
2. $\mathfrak {M}$ is *B*-regular if $\mathfrak {M}$ satisfies (2.9), (2.11) and (2.16).

We write [weakly regular$]=$weakly *R*-regular, weakly *B*-regular. Clearly, every [weakly regular] weight matrix $\mathfrak {M}$ is [semiregular].

#### Remark 2.11

If $\omega$ is a weight function then the weight matrix $\mathfrak {W}$ associated to $\omega$ consists of the weight sequences $\textbf{W}^\lambda$, $\lambda >0$, which are given by

$$\begin{aligned} W_k^\lambda =e^{\frac{1}{\lambda }\varphi _\omega ^*(\lambda k)}. \end{aligned}$$

It is easy to see that $\mathcal {E}^{[ \omega ]} ( U )=\mathcal {E}^{[ \mathfrak {W} ]} ( U )$ as topological vector spaces. If $\omega (t)=o(t)$ then $\mathfrak {W}$ is *R*- and *B*-semiregular. Furthermore, (2.10) and (2.11) hold both for $\mathfrak {W}$, cf. [34].

When $\omega$ is concave then there exists a weight matrix $\mathfrak {T}$ such that $\mathfrak {T}\{\approx \}\mathfrak {W}$ and $\mathfrak {T}(\approx )\mathfrak {W}$ and moreover $\mathfrak {T}$ satisfies (2.16); in fact, every weight sequence $\textbf{T}\in \mathfrak {T}$ satisfies (2.2), see [36, Proposition 3]. Moreover, $\mathfrak {T}$ satisfies (2.9), since condition (2.9) is clearly invariant under $[\approx ]$. Thus $\mathcal {E}^{[ \omega ]} ( \Omega )=\mathcal {E}^{[ \mathfrak {T} ]} ( \Omega )$ and $\mathfrak {T}$ is weakly *R*- and weakly *B*-regular since the conditions (2.10) and (2.11) are also invariant under the equivalence relations $\{\approx \}$ and $(\approx )$, respectively.

### Ultradifferentiable vectors associated to weight matrices

#### Definition 2.12

Let $\textsf{P}=\{P_1,\dotsc ,P_\ell \}$ be a system of smooth differential operators in *U*, $d_j$ be the order of $P_j$ for $j=1,\dotsc ,\ell$, $u\in \mathcal {E}(U)$ and $\mathfrak {M}$ be a weight matrix.

1. Assume that $a_{j\lambda }\in \mathcal {B}^{\{ \mathfrak {M} \}} ( U )$ for all ${|\lambda |}\le d_j$ and every $j\in \{1,\dotsc ,\ell \}$. Then we say that *u* is a global vector of class $\{\mathfrak {M}\}$ if there are $\textbf{M}\in \mathfrak {M}$ and $C,h>0$ such that
  2.17 $$\begin{aligned} \Vert {P^\tau u} \Vert _{L^2(U)}\le Ch^{|\tau |} M_{d_\tau } \end{aligned}$$
  for all $\tau \in \{1,\dotsc ,\ell \}^k$, $d_\tau =\sum _{j=1}^k d_{\tau _j}$ and all $k\in \mathbb {N}_0$.
2. When $a_{j\lambda }\in \mathcal {B}^{( \mathfrak {M} )} ( U )$ for all ${|\lambda |}\le d_j$ and every $j\in \{1,\dotsc ,\ell \}$ then *u* is a global vector of class $(\mathfrak {M})$ if for all $\textbf{M}\in \mathfrak {M}$ and all $h>0$ there is a constant $C>0$ such that (2.17) holds for all $\tau \in \{1,\dotsc ,\ell \}^k$ and every $k\in \mathbb {N}_0$.
3. Assume that $a_{j\lambda }\in \mathcal {E}^{\{ \mathfrak {M} \}} \left( U \right)$ for all ${|\lambda |}\le d_j$ and every $j\in \{1,\dotsc ,\ell \}$. Then we say that *u* is a (local) vector of class $\{\mathfrak {M}\}$ if for all $V\Subset U$ there are $\textbf{M}\in \mathfrak {M}$ and $C,h>0$ such that
  2.18 $$\begin{aligned} \Vert {P^\alpha u} \Vert _{L^2(V)}\le Ch^{|\alpha |}M_{|\alpha |}\end{aligned}$$
  for $$\alpha \in \{1,\dotsc ,\ell \}^k$$ and $$k\in \mathbb {N}_0$$.
4. When $a_{j\lambda }\in \mathcal {E}^{( \mathfrak {M} )} ( U )$ for all ${|\lambda |}\le d_j$ and every $j\in \{1,\dotsc ,\ell \}$ then *u* is a local vector of class $(\mathfrak {M})$ if for all $V\Subset U$, all $\textbf{M}\in \mathfrak {M}$ and all $h>0$ there is a constant $C>0$ such that (2.18) holds for all $\alpha \in \{1,\dotsc ,\ell \}^k$ and every $k\in \mathbb {N}_0$.

We denote the space of global vectors of class $[\mathfrak {M}]$ in *U* by $\mathcal {B}^{[ \mathfrak {M} ]} ( U ; \textsf{P} )$ and $\mathcal {E}^{[ \mathfrak {M} ]} ( U ; \textsf{P} )$ is the space of all local vectors of class $[\mathfrak {M}]$.

#### Proposition 2.13

Let $\mathfrak {M}$ be a weight matrix and $\textsf{P}=\{P_1,\dotsc ,P_\ell \}$ be a system of smooth differential operators in an open set $U\subseteq {\mathbb {R}}^n$. Then the following holds:

1. If the coefficients of the operators $P_j$ are all in $\mathcal {E}^{[ \mathfrak {M} ]} ( U )$ then $\mathcal {E}^{[ \mathfrak {M} ]} ( U )\subseteq \mathcal {E}^{[ \mathfrak {M} ]} ( U ; \textsf{P} )$.
2. If *U* is bounded and the coefficients of the operators $P_j$ are all in $\mathcal {B}^{[ \mathfrak {M} ]} ( U )$ then $\mathcal {B}^{[ \mathfrak {M} ]} ( U )\subseteq \mathcal {B}^{[ \mathfrak {M} ]} ( U ; \textsf{P} )$.

#### Proof

Suppose that $Q(x,D)=\sum _{{|\alpha |}\le d}b_{\alpha }D^\alpha$ is a partial differential operator with coefficients $b_\alpha \in \mathcal {B}^{\{ \mathfrak {M} \}} ( U )$ and $f\in \mathcal {B}^{\{ \mathfrak {M} \}} ( U )$. Then we can assume that there exist some $h>0$ and $\textbf{M}\in \mathfrak {M}$ such that $b_\alpha ,f\in \mathcal {B}_{ \textbf{M} , h }(U)$. It follows that

$$\begin{aligned} \begin{aligned} \Vert {D^\beta Qf} \Vert _{L^2(U)}&\le C\sum _{{|\alpha |}\le d}\sum _{\gamma \le \beta }\left( {\begin{array}{c}\beta \\ \gamma \end{array}}\right) h^{{|\beta |}-{|\gamma |}}M_{{|\beta |}-{|\gamma |}}h^{{|\gamma |}+{|\alpha |}}M_{{|\gamma |}+{|\alpha |}}\\&\le C^\prime (h^\prime )^{{|\beta |}+d} M_{{|\beta |}+d} \end{aligned} \end{aligned}$$

for some constants $C^\prime >0$ and $h^\prime >0$. From this estimate we can conclude that $\mathcal {B}^{\{ \mathfrak {M} \}} ( U )\subseteq \mathcal {B}^{\{ \mathfrak {M} \}} \left( U; \textsf{P} \right)$.

In the Beurling case we have that $b_\alpha ,f\in \mathcal {B}^{( \mathfrak {M} )} ( U )=\bigcap _{\textbf{M}\in \mathfrak {M}}\mathcal {B}^{( \textbf{M} )} ( U )$. We define a sequence $\textbf{L}$ by

$$\begin{aligned} L_k=\max \left\{ \sup _{x\in U} |D^\beta f(x)|;\sup _{x\in U} |D^\beta b_\alpha (x)|:\; {|\alpha |}\le d,\,{|\beta |}\le k\right\} . \end{aligned}$$

If $\textbf{M}\in \mathfrak {M}$ is arbitrary then $\textbf{L}\lhd \textbf{M}$. According to the proof of [20, Lemma 2.2] there is a weight sequence $\textbf{N}$ such that $\textbf{L}\le \textbf{N}\lhd \textbf{M}$. Thus $b_\alpha ,f\in \mathcal {B}_{ \textbf{N} , h }(U)$ for some *h*. The estimate above gives

$$\begin{aligned} \Vert {D^\beta Qf} \Vert _{L^2(U)}\le C^\prime (h^\prime )^{{|\beta |}+d}N_{{|\beta |}+d} \end{aligned}$$

for some $C^\prime ,h^\prime >0$. Hence for all $h>0$ there is a constant $C>0$ such that

$$\begin{aligned} \Vert {D^\beta Qf} \Vert _{L^2(U)}\le Ch^{{|\beta |}+d}M_{{|\beta |}+d}. \end{aligned}$$

Since $\textbf{M}\in \mathfrak {M}$ was chosen arbitrarily we conclude that $\mathcal {B}^{( \mathfrak {M} )} ( U )\subseteq \mathcal {B}^{ (\mathfrak {M}) } ( U ; \textsf{P} )$.

The local case follows analogously. $\square$

We can now state our main theorem:

#### Theorem 2.14

Let $\mathfrak {M}$ be a [weakly regular] weight matrix and $\textsf{P}=\{P_1,\dotsc ,P_\ell \}$ be an elliptic system of differential operators of class $[\mathfrak {M}]$ in $\Omega$. Then

$$\begin{aligned} \mathcal {E}^{[ \mathfrak {M} ]} ( \Omega ; \textsf{P} )=\mathcal {E}^{[ \mathfrak {M} ]} ( \Omega ). \end{aligned}$$

#### Remark 2.15

Note that we can assume that all the operators $P_j\in \textsf{P}$ in Theorem 2.14 have the same order. Indeed, if $d_j$ denotes the order of $P_j$ for all $j\in \{1,\dotsc ,\ell \}$ then we set $d^\prime _j=\prod _{i\ne j}d_i$ and $Q_j=P_j^{d^\prime _j}$. Then all $Q_j$ are of order $d=\prod _j d_j$ and it is easy to see that the system $\textsf{Q}=\{Q_1,\dotsc ,Q_\ell \}$ is elliptic if and only if $\textsf{P}$ is elliptic. Furthermore $\mathcal {E}^{[ \mathfrak {M} ]} ( \Omega ; \textsf{P} )\subseteq \mathcal {E}^{[ \mathfrak {M} ]} ( \Omega ; \textsf{Q} )$ for all weight matrices $\mathfrak {M}$. Hence, Theorem 2.14 will be proven if we show $\mathcal {E}^{[ \mathfrak {M} ]} ( \Omega ; \textsf{Q} )\subseteq \mathcal {E}^{[ \mathfrak {M} ]} ( \Omega )$.

## The fundamental estimate

By Remark 2.15 we will assume in this and the next section that all operators $P_j\in \textsf{P}$ are of the same order $d\in \mathbb {N}$. We will also denote the open ball of radius *R* centered at a point $x\in {\mathbb {R}}^n$ by *B*(*x*, *R*).

We follow closely the structure of the proof given in [7] (see also [9]).

Beginning with a well-known a-priori estimate for elliptic systems of smooth operators of equal order *d* (see e.g. [2]), i.e. for all $U\Subset \Omega$ there is a constant $C>0$ such that

$$\begin{aligned} \Vert {u} \Vert _{H^k(U)}\le C\left( \sum _{j=1}^\ell \Vert {P_ju} \Vert _{H^{k-d}(U)} +\Vert {u} \Vert _{L^2(U)}\right) ,\qquad \quad u\in \mathcal {D}(U),\; k=0,\dotsc ,d, \end{aligned}$$

we can deduce two other estimates following the arguments in [7]:

### Proposition 3.1

(cf. [7, Proposition I-2]) Let $\textsf{P}$ be an elliptic system of differential operators with equal order *d* and smooth coefficients in $\Omega$ and let $W^\prime \Subset W\Subset \Omega$ be open sets. Then there exists a constant $C>0$ such that

3.1 $$\begin{aligned} \Vert {u} \Vert _{H^d(W^\prime )}\le C\left( \sum _{j=1}^\ell \Vert {P_ju} \Vert _{L^2(W)} +\Vert {u} \Vert _{L^2(W)}\right) \end{aligned}$$

for all $$u\in \mathcal {E}(\Omega )$$.

### Proposition 3.2

(cf. [7, Proposition I-3]) Let $\textsf{P}$ be as in Proposition 3.1, $x\in \Omega$ and $R,R_1>0$ with $R<R_1$ such that $B(x;R_1)\Subset \Omega$. For $\rho < R$ write $W[\rho ]=B(x;R-\rho )$. Then there exists a constant $C>0$ such that for all $u\in \mathcal {E}(\Omega )$, for all $\alpha \in \mathbb {N}_0^n$ with ${|\alpha |}\le d$ and for all $\rho ,\rho ^\prime >0$ with $\rho +\rho ^\prime <R$ we have

3.2 $$\begin{aligned} \rho ^d\Vert {D^\alpha u} \Vert _{L^2(W[\rho +\rho ^\prime ])}\le C\left( \rho ^d\sum _{j=1}^\ell \Vert {P_ju} \Vert _{L^2(W[\rho ^\prime ])} +\sum _{{|\beta |}\le d-1}\rho ^{|\beta |}\Vert {D^\beta u} \Vert _{L^2(W[\rho ^\prime ])} \right) .\nonumber \\ \end{aligned}$$

We are now in the position to formulate and prove the main estimate which will be used in the proof of Theorem 2.14. Note that if $\rho _1<\rho _2<R$ then $W[\rho _2]\Subset W[\rho _1]$. We may also set $W[\rho ]=\emptyset$ if $\rho >R$. Moreover, we recall also that for a weight sequence $\textbf{M}$ we have defined the auxiliary sequence $(\Theta _k)_k$ by $\Theta _k=k\root k \of {m_k}$.

### Proposition 3.3

Let $\textbf{M}$ be a sequence with $M_0=1\le M_1$ satisfying (2.1) and (2.2), $x\in \Omega$, $R<R_1\le 1$ such that $W=B(x,R_1)\Subset \Omega$ and assume $\textsf{P}=\{P_1,\dotsc ,P_\ell \}$ is an elliptic system of smooth differential operators $P_j=\sum _{{|\lambda |}\le d} a_{j\lambda }D^\lambda$ on $\Omega$ such that $a_{j\lambda }\vert _W\in \mathcal {B}^{\{ \textbf{M} \}} ( W )$. Then there exists a constant $A>0$ such that for all $0<\rho <R$, all $u\in \mathcal {E}(\Omega )$ and all $k\in \mathbb {N}$ we have that

3.3 $$\begin{aligned} \rho ^{{|\alpha |}}\Vert {D^\alpha u} \Vert _{L^2(W[\Theta _{{|\alpha |}}\rho ])} \le A^{{|\alpha |}+1} S_k(u) \end{aligned}$$

where $${|\alpha |}\le dk$$ and

$$\begin{aligned} S_k(u)=\sum _{\sigma =1}^{k}\rho ^{(\sigma -1)d} \sum _{\tau \in \{1,\dotsc ,\ell \}^{\sigma }} \Vert {P^\tau u} \Vert _{L^{2}(W)}+\Vert {u} \Vert _{L^2(W)}. \end{aligned}$$

### Proof

We begin by observing that $S_{k}(u)\le S_{k+1}(u)$ and

$$\begin{aligned} \sum _{j=1}^\ell \rho ^d S_k \left( P_ju \right) \le S_{k+1}(u) \end{aligned}$$

for all $k\in \mathbb {N}$ since $\rho \le 1$ by assumption.

Furthermore there exists a constant $H>0$ such that

$$\begin{aligned} \Vert {D^\gamma a_{j,\lambda }} \Vert _{L^2(W)}\le H^{{|\gamma |}+1} M_{|\gamma |}\end{aligned}$$

for all $\gamma \in \mathbb {N}_0^n$ and $\lambda \in \mathbb {N}_0^n$ with ${|\lambda |}\le d$ and every $j\in \{1,\dotsc ,\ell \}$.

We are going to prove (3.3) by induction in *k*. To begin with, (3.1) implies that

$$\begin{aligned} \Vert {D^\alpha u} \Vert _{L^2(W[0])}\le A\left( \sum _{j=1}^\ell \Vert {P_ju} \Vert _{L^2(W)} +\Vert {u} \Vert _{L^2(W)} \right) \end{aligned}$$

for ${|\alpha |}\le d$ which gives (3.3) for $k=1$ since $\rho \le 1$ and without loss of generality we can assume that $A\ge 1$.

Now let $\alpha \in \mathbb {N}_0^n$ be such that $dk<{|\alpha |}\le d(k+1)$ and assume that (3.3) has been shown for all $\beta \in \mathbb {N}_0^n$ with ${|\beta |}\le {|\alpha |}-1$. We put $\alpha =\alpha _0+\alpha ^\prime$ with $|\alpha _0|=d$. If we replace in (3.2) $\rho ^\prime$ by $(\Theta _{{|\alpha |}} -1)\rho$, $\alpha$ by $\alpha _0$ and *u* by $D^{\alpha ^\prime }u$, then we obtain

$$\begin{aligned} \rho ^{{|\alpha |}}\Vert {D^\alpha u} \Vert _{L^2(W[\Theta _{{|\alpha |}}\rho ])}\le & {} C\left\{ \rho ^{{|\alpha |}}\sum _{j=1}^\ell \Vert {P_j\bigl (D^{\alpha ^\prime }u\bigr )} \Vert _{L^2(W[(\Theta _{{|\alpha |}}-1)\rho ])}\right. \\{} & {} \qquad \qquad \left. +\sum _{{|\beta |}\le d-1}\rho ^{{|\alpha |}-d+{|\beta |}} \Vert {D^{\beta +\alpha ^\prime }u} \Vert _{L^2(W[(\Theta _{|\alpha |}-1)\rho ])} \right\} . \end{aligned}$$

In the following we use the notation $\beta <\alpha$ if $\beta \le \alpha$ and $\beta \ne \alpha$ for $\alpha ,\beta \in \mathbb {N}_0^n$. We observe that

$$\begin{aligned} D^{\alpha ^\prime }(P_j u)-P_j\Bigl (D^{\alpha ^\prime }u\Bigr ) =\sum _{{|\lambda |}\le d} \sum _{\begin{array}{c} \gamma <\alpha ^\prime \end{array}} \left( {\begin{array}{c}\alpha ^\prime \\ \gamma \end{array}}\right) D^{\alpha ^\prime -\gamma }a_{j\lambda } D^{\lambda +\gamma }u \end{aligned}$$

can be estimated by

3.4 $$\begin{aligned}&\Vert {D^{\alpha ^\prime }(P_j u) -P_j\left( D^{\alpha ^\prime }u\right) } \Vert _{L^2(W[(\Theta _{{|\alpha |}}-1)\rho ])} \end{aligned}$$

3.5 $$\begin{aligned}&\quad \le \sum _{{|\lambda |}\le d} \sum _{\gamma <\alpha ^\prime } \left( {\begin{array}{c}|\alpha ^\prime |\\ {|\gamma |}\end{array}}\right) H^{|\alpha ^\prime -\gamma |+1}M_{|\alpha ^\prime -\gamma |} \bigl (\Theta _{|\alpha ^\prime |}\rho \bigr )^{-|\alpha ^\prime -\gamma |} \Vert {D^{\beta +\gamma }u} \Vert _{L^2(W[(\Theta _{{|\alpha |}}-1)\rho ])} \end{aligned}$$

since if $\Theta _{|\alpha ^\prime |}\rho >R$ then $W[(\Theta _{{|\alpha |}}-1)\rho ]=\emptyset$, because $\Lambda _{{|\alpha |}}-\Lambda _{|\alpha ^\prime |}\ge d$ by (2.6). Now note that $\Lambda _k\le \Theta _k$. Furthermore, using also the fact that $$\left( {\begin{array}{c}k\\ j\end{array}}\right) \le k^j/(k!)$$ for $0\le j\le k$ we conclude that

3.6 $$\begin{aligned} \begin{aligned} \left( {\begin{array}{c}|\alpha ^\prime |\\ |\alpha ^\prime -\gamma |\end{array}}\right) M_{|\alpha ^\prime -\gamma |}\Theta _{|\alpha ^\prime |}^{ -|\alpha ^\prime -\gamma |}&\le \left( {\begin{array}{c}|\alpha ^\prime |\\ |\alpha ^\prime -\gamma |\end{array}}\right) |\alpha ^\prime -\gamma |! m_{|\alpha ^\prime -\gamma |} |\alpha ^\prime |^{-|\alpha ^\prime -\gamma |} \left( m_{|\alpha ^\prime |^{1/|\alpha ^\prime |}}\right) ^{-|\alpha ^\prime -\gamma |}\\&\le \left( \frac{m_{|\alpha ^\prime -\gamma |}^{1/|\alpha ^\prime -\gamma |}}{ m_{|\alpha ^\prime |}^{1/|\alpha ^\prime |}}\right) \prod _{j=1}^{|\alpha ^\prime -\gamma |}\frac{|\gamma |+j}{|\alpha ^\prime |} \le 1 \end{aligned} \end{aligned}$$

since $\root k \of {m_k}$ is increasing. Therefore

$$\begin{aligned} \rho ^{{|\alpha |}}\Vert {D^\alpha u} \Vert _{L^2(W_{\Theta _{{|\alpha |}}\rho })}\le & {} C\left\{ \rho ^{{|\alpha |}}\sum _{j=1}^\ell \Vert {D^{\alpha ^\prime }\bigl (P_ju\bigr )} \Vert _{L^2(W[\Theta _{|\alpha ^\prime |}\rho ])} \right. \\{} & {} \qquad +\sum _{{|\lambda |}\le d}\sum _{\gamma <\alpha ^\prime } H^{|\alpha ^\prime |-{|\gamma |}+1}\rho ^{{|\alpha |}-|\alpha ^\prime |+{|\gamma |}} \Vert {D^{\lambda +\gamma }u} \Vert _{L^2(W[\Theta _{(d+{|\gamma |})}\rho ])}\\{} & {} \qquad \left. +\sum _{{|\beta |}\le d-1}\rho ^{{|\alpha |}-d+{|\beta |}} \Vert {D^{\beta +\alpha ^\prime }u} \Vert _{L^2(W[\Theta _{{|\beta |}+|\alpha ^\prime |}\rho ])} \right\} , \end{aligned}$$

since $W[(\Theta _k-1)\rho ]\subseteq W[\Theta _\ell \rho ]$ by (2.6) for all $\ell <k$. Now, the induction hypothesis implies the following estimates:

$$\begin{aligned} \rho ^{{|\alpha |}}\sum _{j=1}^\ell \Vert {D^{\alpha ^\prime } \bigl (P_ju\bigr )} \Vert _{L^2(W[\Theta _{|\alpha ^\prime |}\rho ])}&\le \rho ^d A^{|\alpha ^\prime |+1}\sum _{j=1}^\ell S_k(P_ju)\le A^{|\alpha ^\prime |+1} S_{k+1}(u),\\ \sum _{\begin{array}{c} {|\lambda |}\le d\\ \gamma<\alpha ^\prime \end{array}} H^{|\alpha ^\prime |-{|\gamma |}+1}\rho ^{d+{|\gamma |}} \Vert {D^{\lambda +\gamma }u} \Vert _{L^2(W[\Theta _{(d+{|\gamma |})}\rho ])}&\le \sum _{\begin{array}{c} {|\lambda |}\le d\\ \gamma <\alpha ^\prime \end{array}} H^{|\alpha ^\prime |-{|\gamma |}+1} A^{d+{|\gamma |}+1}S_{k+1}(u),\\ \sum _{{|\beta |}\le d-1}\rho ^{{|\alpha |}-d+{|\beta |}} \Vert {D^{\beta +\alpha ^\prime }u} \Vert _{L^2(W[\Theta _{{|\beta |}+|\alpha ^\prime |}\rho ])}&\le \sum _{{|\beta |}\le d-1}A^{|\alpha ^\prime |+{|\beta |}+1}S_{k+1}(u). \end{aligned}$$

Hence we have obtained that

3.7 $$\begin{aligned} \begin{aligned}&\rho ^{{|\alpha |}}\Vert {D^\alpha u} \Vert _{L^2(W[\Theta _{{|\alpha |}}\rho ])} \le A^{{|\alpha |}+1}S_{k+1}(u)\\&\qquad \times \left\{ CA^{-d}+C\sum _{{|\lambda |}\le d} \sum _{\gamma <\alpha ^\prime } H^{|\alpha ^\prime |-{|\gamma |}+1} A^{{|\gamma |}-|\alpha ^\prime |} +C\sum _{{|\beta |}\le d-1}A^{{|\beta |}-d}\right\} . \end{aligned} \end{aligned}$$

Since

$$\begin{aligned} C\sum _{{|\lambda |}\le d} \sum _{\gamma <\alpha ^\prime } H^{|\alpha ^\prime |-{|\gamma |}+1} A^{{|\gamma |}-|\alpha ^\prime |} \le Cd^nH^2A^{-1}\sum _{\beta \in \mathbb {N}^n_0}\bigl (HA^{-1}\bigr )^{|\beta |}, \end{aligned}$$

we are able to choose *A* large enough and independent of $\alpha$ and $\rho$ so that the bracket on the right-hand side of (3.7) is $\le 1$. $\square$

## Proof of Theorem 2.14

### The Roumieu case

Let $\mathfrak {M}$ be an *R*-regular weight matrix and $u\in \mathcal {E}^{\{ \mathfrak {M} \}} \left( \Omega ; \textsf{P} \right)$. We have to prove that for all $x\in \Omega$ there is a neighborhood $U\Subset \Omega$ of *x* such that $u\vert _U\in \mathcal {B}^{\{ \mathfrak {M} \}} ( U )$.

Therefore we fix $x_0\in \Omega$ and choose $R_1\le 1$ such that $W=B(x_0;R_1)\Subset \Omega$. Then by assumptation there are a weight sequence $\textbf{M}\in \mathfrak {M}$, satisfying (2.1) and (2.2), and constants $C,h>0$ such that

$$\begin{aligned} \Vert {P^\tau u} \Vert _{L^2(W)}\le Ch^k M_{dk} \end{aligned}$$

for all $\tau \in \{1,\dotsc ,\ell \}^k$ and all $k\in \mathbb {N}$.

We conclude that

$$\begin{aligned} S_k(u)\le C\sum _{\sigma =1}^k\rho ^{(\sigma -1)d}\ell ^\sigma h^\sigma M_{d\sigma }+C \end{aligned}$$

for all $0<\rho <R$ for some $0<R<R_1$.

Hence by (3.3) we have that

$$\begin{aligned} \Vert {D^\alpha u} \Vert _{L^2(W[\mu _{|\alpha |}\rho ])} \le CA^{{|\alpha |}+1}\left( \sum _{\sigma =1}^k\rho ^{d(\sigma -1)-{|\alpha |}}\ell ^\sigma h^\sigma M_{d\sigma }+1\right) \end{aligned}$$

for every $0<\rho < R$, all $\alpha \in \mathbb {N}_0^n$ with $d(k-1)<{|\alpha |}\le dk$ for $k\in \mathbb {N}$ where $C,A,h>0$ are constants independent of $\rho$, *k* and $\alpha$.

Now choose some $R^\prime$ with $0<R^\prime<R<R_1<1$. In particular $R-R^\prime <1$ and we set

$$\begin{aligned} \rho =\frac{R-R^\prime }{e\Lambda _{dk}}. \end{aligned}$$

Thus, we have for $d(k-1)\le {|\alpha |}\le dk$ that $dk\le {|\alpha |}+d$ and therefore

$$\begin{aligned} \begin{aligned} M_{d\sigma }\rho ^{d(\sigma -1)-{|\alpha |}}&=\left( \Lambda _{d\sigma }\right) ^{d\sigma } \left( \frac{R-R^\prime }{e}\right) ^{d(\sigma -1)-{|\alpha |}} \left( \Lambda _{dk}\right) ^{{|\alpha |}-d(\sigma -1)}\\&\le \left( \frac{R-R^\prime }{e}\right) ^{d(\sigma -1)-{|\alpha |}}\Lambda _{dk}^{{|\alpha |}+d}\\&\le R_2^{{|\alpha |}-d(\sigma -1)}M_{{|\alpha |}+d} \end{aligned} \end{aligned}$$

where $$R_2=e(R-R^\prime )^{-1}>1$$.

Now, note that the sequence $\Theta _k$ is strictly increasing and the Stirling formula implies that

$$\begin{aligned} \frac{1}{e}\le \frac{\Theta _k}{e\Lambda _k}= \frac{k}{e(k!)^{1/k}} \le \frac{1}{(2\pi k)^{1/2k}}\le 1 \end{aligned}$$

for all $k\in \mathbb {N}$. Thence we have the following estimate

$$\begin{aligned} \begin{aligned} R-\Theta _{|\alpha |}\rho&\ge R-\Theta _{dk}\rho \\&= R\left( 1-\frac{\Theta _{dk}}{e\Lambda _{dk}}\right) +\frac{\Theta _{dk}}{e\Lambda _{dk}}R^\prime \\&\ge e^{-1}R^\prime \end{aligned} \end{aligned}$$

and therefore $U=B(x_0,e^{-1}R^\prime )\subseteq W[\Lambda _{|\alpha |}\rho ]$. Thus we can, if we enlarge *h* when necessary, estimate that

$$\begin{aligned} \begin{aligned} \Vert {D^\alpha u} \Vert _{L^2(U)}&\le CA^{{|\alpha |}+1}\left( \sum _{\sigma =1}^kR_2^{{|\alpha |}-d(\sigma -1)}\ell ^\sigma h^\sigma M_{{|\alpha |}+d}+1\right) \\&\le CA^{{|\alpha |}+1}(\ell h)^{({|\alpha |}+d)/d} R_2^{|\alpha |}M_{{|\alpha |}+d} \sum _{\sigma =0}^k1\\ \end{aligned} \end{aligned}$$

for every $k\in \mathbb {N}$ and all $\alpha \in \mathbb {N}_0^n$ with $d(k-1)<{|\alpha |}\le dk$.

Since *d* does not depend on $\alpha$ or *k* we have by (2.12) that there is a weight sequence $\textbf{M}^\prime$ and constants $C_1,h_1$ such that

$$\begin{aligned} \Vert {D^\alpha u} \Vert _{L^2(U)}\le C_1 h_1^{{|\alpha |}} M^\prime _{{|\alpha |}} \end{aligned}$$

for all $\alpha \in \mathbb {N}_0^n$. Thus $u\in \mathcal {E}^{\{ \mathfrak {M} \}} \left( \Omega \right)$ by Remark 2.9(3).

### The Beurling case

Now we assume that $\mathfrak {M}$ is weakly *B*-regular and $u\in \mathcal {E}^{ (\mathfrak {M}) } ( U ; \textsf{P} )$. Note that

$$\begin{aligned} \mathcal {E}^{( \mathfrak {M} )} ( \Omega )=\bigcap _{\textbf{M}\in \mathfrak {M}}\mathcal {E}^{( \textbf{M} )} ( \Omega ),\qquad \mathcal {E}^{ (\mathfrak {M}) } ( \Omega ; \textsf{P} )=\bigcap _{\textbf{M}\in \mathfrak {M}}\mathcal {E}^{ (\textbf{M}) } ( \Omega ; \textsf{P} ). \end{aligned}$$

Thus we consider first the case where $u\in \mathcal {E}^{ (\textbf{M}) } ( \Omega ; \textsf{P} )$ and $a_{j\lambda }\in \mathcal {E}^{( \textbf{M} )} ( \Omega )$, $1\le j\le \ell$, ${|\lambda |}\le d$, with $\textbf{M}$ being a weight sequence for which (2.1) and (2.5) hold. We fix $x\in \Omega$ and let $0<R<R_1\le 1$ be such that $W=B(x;R_1)\Subset \Omega$. We define a sequence $\textbf{L}$ by setting

$$\begin{aligned} \begin{aligned} L_k&=\max \Bigl \{k!;\;\sup _{x\in W} |D^\alpha a_{j,\lambda }|:\,{|\alpha |}\le k,{|\lambda |}\le d, j\in \{1,\dotsc ,\ell \};\\&\qquad \qquad \qquad \qquad \qquad \Vert {P^\tau u} \Vert _{L^2(W)}:\, \tau \in \{1,\dotsc ,\ell \}^{\nu }, \nu \le \tfrac{k}{d} \Bigr \}. \end{aligned} \end{aligned}$$

According to Lemma 2.5 there is a sequence $\textbf{N}$ with $N_0=1\le N_1$ satisfying (2.1) and (2.5) such that $\textbf{L}\preceq \textbf{N}\lhd \textbf{M}$. Hence $u\in \mathcal {B}^{\{ \textbf{N} \}} \left( W; \textsf{P} \right)$ and $a_{j\lambda }\in \mathcal {B}^{\{ \textbf{N} \}} ( W )$. It follows that we can apply Proposition 3.3 and obtain that there is a constant *A* such that for all $k\in \mathbb {N}$ and every $\alpha$ with $d(k-1)<{|\alpha |}\le dk$ we have

$$\begin{aligned} \rho ^{{|\alpha |}}\Vert {D^\alpha u} \Vert _{L^2(W_{\nu _{|\alpha |}\rho })}\le A^{{|\alpha |}+1}S_k(u) \end{aligned}$$

where $S_k(u)$ is as in Proposition 3.3 with $\textbf{M}$ replaced by $\textbf{N}$ and $0<\rho <R$ is chosen arbitrarily but fixed. Since (2.5) still implies that $\textbf{N}$ and $\root k \of {N_k}$ are increasing, the arguments in the previous subsection yield that there are a neighborhood *U* of $x_0$ and constants $C_1,h_1>0$ such that

$$\begin{aligned} \Vert {D^\alpha u} \Vert _{L^2(U)}\le C_1h_1^{|\alpha |}N_{{|\alpha |}+d} \end{aligned}$$

for all $k\in \mathbb {N}$ and all $d(k-1)<{|\alpha |}\le dk$. Since $\textbf{N}\lhd \textbf{M}$ we conclude that for all $h>0$ there is a constant $C>0$ such that

$$\begin{aligned} \Vert {D^\alpha u} \Vert _{L^2(U)}\le Ch^{{|\alpha |}} M_{{|\alpha |}+d}. \end{aligned}$$

But $\textbf{M}\in \mathfrak {M}$ has been chosen arbitrarily and therefore we obtain the above estimate for all $\textbf{M}\in \mathfrak {M}$ if $u\in \mathcal {E}^{ (\mathfrak {M}) } ( \Omega ; \textsf{P} )$. Now we can employ (2.13) to conclude that for all weight sequences $\textbf{M}^\prime$ and $h_1>0$ there is a constant $C_1>0$ such that

$$\begin{aligned} \Vert {D^\alpha u} \Vert _{L^2(U)}\le C_1h_1^{{|\alpha |}} M^\prime _{{|\alpha |}+d}. \end{aligned}$$

Applying Remark 2.9(3) we observe that $u\in \mathcal {E}^{( \mathfrak {M} )} ( \Omega )$.

## Remarks

### Elliptic regularity in ultradifferentiable classes

Let $\mathfrak {M}$ be a weight matrix and $\textsf{P}$ be an elliptic system of differential operators with $\mathcal {E}^{[ \mathfrak {M} ]} ( \Omega )$-coefficients. We note that in that case instead of $u\in \mathcal {E}(\Omega )$ we can just assume that $u\in \mathcal {D}^\prime (\Omega )$ in Definition 2.12 by the subellipticity of the elliptic system $\textsf{P}$, cf. [9] or [37].^3^ This allows us to deduce results on ultradifferentiable hypoellipticity from Theorem 2.14.

#### Definition 5.1

Let $\mathfrak {M}$ be a weight matrix and $\textsf{P}=\{P_1,\dotsc ,P_\ell \}$ be a system of differential operators with $\mathcal {E}^{[ \mathfrak {M} ]} ( \Omega )$-coefficients. We say that $\textsf{P}$ is $[\mathfrak {M}]$-hypoelliptic if for any open $U\subseteq \Omega$ and all $u\in \mathcal {D}^\prime (U)$ the fact that $P_ju\in \mathcal {E}^{[ \mathfrak {M} ]} ( U )$, $j=1,\dotsc ,\ell$, implies that $u\in \mathcal {E}^{[ \mathfrak {M} ]} ( U )$.

#### Theorem 5.2

Let $\mathfrak {M}$ be a [weakly regular] weight matrix and $\textsf{P}$ be an elliptic system of operator of class $[\mathfrak {M}]$ in $\Omega$. Then $\textsf{P}$ is $[\mathfrak {M}]$-hypoelliptic in $\Omega$.

It is worthwile to compare the conditions on the weight matrix in Theorem 5.2 with the hypothesis needed in the microlocal regularity results given in [19, Section 7]. For simplicity we restrict our discussion to Denjoy–Carleman classes. In [19] we proved the following result:

#### Theorem 5.3

([19, Theorem 7.1 & Theorem 7.4]) Let $\textbf{M}$ be a weight sequence satisfying (1.3), (1.4) and (1.8). Then for any differential operator with coefficients in $\mathcal {E}^{[ \textbf{M} ]} ( \Omega )$ we have that

$$\begin{aligned} {{\,\textrm{WF}\,}}_{[\textbf{M}]}u\subseteq {{\,\textrm{WF}\,}}_{[\textbf{M}]}Pu\cup {{\,\textrm{Char}\,}}P \end{aligned}$$

for all $$\mathcal {D}^\prime (\Omega )$$.

Here ${{\,\textrm{WF}\,}}_{[\textbf{M}]}u$ denotes the ultradifferentiable wavefront set with respect to the weight sequence as defined by [22] for Roumieu classes (for the Beurling case see [19]). Moreover, ${{\,\textrm{Char}\,}}P$ is the characteristic set of the linear differential operator *P*, cf. e.g. [23]. Since $\bigcap _{j=1}^\ell {{\,\textrm{Char}\,}}P_j=\emptyset$ for any elliptic system of differential operators and under our assumptations it holds that ${{\,\textrm{WF}\,}}_{[\textbf{M}]}Pu\subseteq {{\,\textrm{WF}\,}}_{[\textbf{M}]} u$ for all $u\in \mathcal {D}^\prime (\Omega )$ and any differential operator *P* of class $[\textbf{M}]$, cf. [19, Proposition 5.4(7)], we obtain the following corollary from Theorem 5.3.

#### Corollary 5.4

Let $\textbf{M}$ be a weight sequence satisfying (1.3), (1.4) and (1.8) and  be an elliptic system of differential operators with coefficients in $\mathcal {E}^{[ \textbf{M} ]} ( \Omega )$. Then

$$\begin{aligned} {{\,\textrm{WF}\,}}_{[\textbf{M}]}u=\bigcap _{j=1}^\ell {{\,\textrm{WF}\,}}_{[\textbf{M}]}P_ju \end{aligned}$$

for all $$u\in \mathcal {D}^\prime (\Omega )$$.

Corollary 5.4 gives that the system $\textsf{P}$ is $[\textbf{M}]$-hypoelliptic, but the statement is in fact stronger, namely it says that the ultradifferentiable hypoellipticity of $\textsf{P}$ holds on the microlocal level, i.e. $\textsf{P}$ is $[\textbf{M}]$-microhypoelliptic. But as we have discussed in the introduction the assumptations on the weight sequence in Corollary 5.4 are much stricter than the conditions in Theorem 5.2. In particular, Theorem 5.2 holds for the weight sequences $\textbf{N}^q$, $q>1$, given by $N_k=q^{k^2}$, but $\textbf{N}^q$ does not satisfy all the conditions in Corollary 5.4.

### Global Kotake–Narasimhan Theorems

Following [7] we can adapt the proof of Theorem 2.14 to obtain a global theorem of iterates.

Suppose that $\Omega \subseteq {\mathbb {R}}^n$ is an open set with boundary $\partial \Omega$ and $\textsf{P}=\{P_1,\dotsc ,P_\ell \}$ a system of differential operators defined on $\overline{\Omega }$ with principal symbols $p_j(x,\xi )$. We say that the system $\textsf{P}$ is *globally elliptic* in $\overline{\Omega }$ if

1. $\textsf{P}$ is elliptic in the interior, i.e. $\Omega$.
2. For all $x\in \partial X$ the polynomials $p_j(x,\xi )$ have no common *complex* zeros in $\xi \in {\mathbb {C}}^n\setminus \{0\}$.

We obtain

#### Theorem 5.5

Let $\mathfrak {M}$ be a [weakly regular] weight matrix, $\Omega \subseteq {\mathbb {R}}^n$ be a bounded open set with Lipschitzian boundary and $\textsf{P}=\{P_1,\dotsc ,P_\ell \}$ be a globally elliptic system of partial differential operators with coefficients in $\mathcal {B}^{[ \mathfrak {M} ]} ( \Omega )$. Then

$$\begin{aligned} \mathcal {B}^{[ \mathfrak {M} ]} ( \Omega ; \textsf{P} )=\mathcal {B}^{[ \mathfrak {M} ]} ( \Omega ). \end{aligned}$$

The proof is based on a global a-priori estimate for globally elliptic systems $\textsf{P}$ of equal order *d*: There is a constant $C>0$ such that for every $u\in \mathcal {D}(\overline{\Omega })$ and $k=1,\dotsc ,d$ we have

5.1 $$\begin{aligned} \Vert {u} \Vert _{H^k(\Omega )}\le C\left\{ \sum _{j=1}^\ell \Vert {P_ju} \Vert _{H^{k-d}(\Omega )} +\Vert {u} \Vert _{L^2(\Omega )}\right\} , \end{aligned}$$

cf. [4, 37] and also [2]. From this estimate, resp. [7, Propositions I-2 & I-3], which are consequences of (5.1) we can follow the lines of the proof of [7, Theorem 2]. We leave the details to the reader.

Similar to the local case Theorem 5.5 yields results on the global ultradifferentiable hypoellipticity of globally elliptic system, cf. [7] for the Gevrey case. We might also note, that we can directly generalize a characterization for global classes given in [7]. Let now $\textsf{P}=\{P_1,\dotsc ,P_\ell \}$ be a system of differential operators $P_j=P_j(D)$ with constant coefficients. We are going to assume that the system $\textsf{P}$ satisfies the following condition:

5.2 $$\begin{aligned} \text {The set of common complex zeros of the polynomials } P_j(\xi ),\,1\le j\le \ell , \text { is finite}. \end{aligned}$$

Here $P_j(\xi )$ denotes the *full* symbol of the operator $P_j(D)$.

#### Theorem 5.6

Let $\Omega \subseteq {\mathbb {R}}^n$ be a bounded open set with Lipschitzian boundary, $\textsf{P}=\{P_1,\dotsc ,P_\ell \}$ be a system of operators with constant coefficients and $\mathfrak {M}$ be a [weakly regular] weight matrix. Then the following statements are equivalent:

2. The system $\textsf{P}$ satisfies (5.2).

#### Proof

First, assume that (1) holds. We setand denote by $Y_k(\Omega )$ the space $Y(\Omega )$ equipped with the $H^k(\Omega )$-norm, for $k\in \mathbb {N}_0$. It is easy to see that the $Y_k(\Omega )$ are all Banach spaces. Moreover, the identity mapping from $Y_{k+1}(\Omega )$ into $Y_k(\Omega )$ is clearly continuous and therefore an isomorphism. Thus all $H^k(\Omega )$-norms are pairwise equivalent to each other on $Y(\Omega )$.

On the other hand, $Y(\Omega )\subseteq \mathcal {E}(\overline{\Omega })$ is a closed subspace of $\mathcal {E}(\overline{\Omega })$ with the usual topology. The Sobolev embedding theorem implies moreover that the topology of $\mathcal {E}(\overline{\Omega })$ is generated by the system of seminorms . Thus on $Y(\Omega )$ the topologies coming from $\mathcal {E}(\overline{\Omega })$ and $H^k(\Omega )$, $k\in \mathbb {N}_0$ agree. Furthermore, $\Omega$ has only finitely many connected components $\Omega _j$ since $\Omega$ is bounded with Lipschitz boundary and for each two points *x*, *y* in a connected components $\Omega _j$ there is a continuous path $\gamma$ connecting *x* with *y* such that the length of $\gamma$ is smaller than $C_j|x-y|$ where $C_j$ is a constant only depending on $\Omega _j$, see [3]. It follows that $\mathcal {E}(\overline{\Omega })$ is nuclear and therefore also $Y(\Omega )$ according to [31]. Thence $Y(\Omega )$ is a nuclear Banach space and thus $Y(\Omega )$ has to be finite dimensional according to [38].

But if $\xi _0\in {\mathbb {C}}^n$ satisfies $P_j(\xi _0)=0$ for all $1\le j\le \ell$ then the function $u(x)=e^{ix\xi _0}$ is a solution of $P_ju=0$ for all $1\le j\le \ell$. Thence the set of all common complex zeros of the polynomials $P_j(\xi )$, $1\le j\le \ell$, has to be finite.

On the other hand, suppose that (2) is true. Let $\xi ^1,\dots ,\xi ^\nu$ be the common complex zeros of the polynomials $P_j(\xi )$, $1\le j\le \ell$. For each $1\le j\le n$ we consider the polynomial

$$\begin{aligned} Q_j(\xi )=\prod _{\kappa =1}^\nu \left( \xi _j-\xi _j^\kappa \right) \end{aligned}$$

where $\xi =(\xi _1,\dotsc ,\xi _n)$ and $\xi ^\kappa =(\xi ^\kappa _1,\dotsc ,\xi ^\kappa _n)$. Then $Q_j(\xi ^\kappa )=0$, $1\le \kappa \le \nu$, that means that the polynomials $Q_j(\xi )$ vanish on the set of common complex zeros of the polynomials $P_j$, $1\le j\le \ell$. The Nullstellensatz, cf. [28, Theorem IX.1.5], implies that there exists an integer $\rho \ge 1$ such that the polynomials $Q_j^\rho$, $1\le j\le n$, belong to the ideal spanned by the polynomials $P_r$, $1\le r\le \ell$. Thence, there exist polynomials $A_{jr}$ such that

$$\begin{aligned} Q_j^\rho (\xi )=\sum _{r=1}^\ell A_{jr}(\xi )P_r(\xi ),\quad 1\le j\le n. \end{aligned}$$

The polynomials $Q_j^\rho (\xi )$ are of order $\nu \rho$ whose principal part is equal to $(\xi _j)^{\nu \rho }$. Thus 0 is the only complex common zero of these principal parts and therefore  is globally elliptic in $\overline{\Omega }$. Furthermore, if $u\in \mathcal {D}^\prime (\Omega )$ and $P_ju\in \mathcal {B}^{[ \mathfrak {M} ]} ( \Omega )$ for $1\le j\le \ell$ then $Q_j^\rho (D)u\in \mathcal {B}^{[ \mathfrak {M} ]} ( \Omega )$ for $1\le j\le n$. From [10] it follows that $u\in \mathcal {E}(\overline{\Omega })$ and therefore $u\in \mathcal {B}^{[ \mathfrak {M} ]} ( \Omega ; \textsf{Q} )$. Finally, according to Theorem 5.5 we have $u\in \mathcal {B}^{[ \mathfrak {M} ]} ( \Omega )$. $\square$

### Final Remarks

We can ask if the conditions, which we have imposed on the data of the ultradifferentiable class $\mathcal {E}^{[\mathfrak {M}]}$ for the Kotake–Narasimhan Theorem to hold, can be further loosened. For this, we recall that we proved the Theorem of Iterates for $\mathcal {E}^{[\mathfrak {M}]}$ in the case of elliptic operators with analytic coefficients when $\mathfrak {M}$ is [semiregular], cf. [20]. But if $\mathfrak {M}$ is [semiregular] then $\mathcal {E}^{[\mathfrak {M}]}$ is closed under derivation and invariant under composition with analytic mappings by [19]. On the other hand, if the weight matrix $\mathfrak {M}$ is [weakly regular], then $\mathcal {E}^{[\mathfrak {M}]}$ is closed under derivation and invariant under composition with ultradifferentiable mappings of class $[\mathfrak {M}]$. Although we must note that the assumption of weakly regularity is not a priori optimal for this fact to hold, see [34]. However, in the case of Braun-Meise-Taylor classes we have that the space $\mathcal {E}^{[\omega ]}$ is invariant under composition with maps of class $[\omega ]$ if and only if $\omega$ is equivalent to a concave weight function. We observe also that $\mathcal {E}^{[\omega ]}$ is closed under derivation by definition.

All these arguments motivate the following conjecture:

#### Conjecture

Let $\mathcal {U}$ be an ultradifferentiable structure which is closed under derivation and is invariant under composition with mappings of class $\mathcal {U}$. Then the Kotake-Narashiman Theorem holds in the class $\mathcal {U}$.

As we have stated, the conjecture is verified for Braun-Meise-Taylor classes, but we claim moreover that the same is true for Denjoy–Carleman classes. Recall from [34] that a Denjoy–Carleman class $\mathcal {E}^{[ \textbf{M} ]} ( \Omega )$ which contains $\mathcal {A}(\Omega )$ (cf. Remark 2.4) and is closed under derivation, i.e. satisfies (1.5), is closed under composition with mappings of class $[\textbf{M}]$ if and only if the sequene $\root k \of {m_k}$ is *almost increasing*, that is

5.3 $$\begin{aligned} \;\,\exists \,C>0:\quad \root j \of {m_j}\le C\root k \of {m_k}\qquad \;\,\forall \,j\le k. \end{aligned}$$

In order to prove our claim let $\textbf{M}$ be a weight sequence such that (5.3) and, if we exclude the analytic case, (2.1) hold. We define a new sequence ${\widetilde{\textbf{M}}}$ by the following procedure.^4^ For $k\in \mathbb {N}$ we set

$$\begin{aligned} \nu _k=C\inf _{\ell \ge k}\root \ell \of {m_\ell } \end{aligned}$$

where $C>0$ is the constant from (5.3). Thence the sequence $\nu _k$ is increasing and $\root k \of {m_k}\le \nu _k\le C\root k \of {m_k}$. We define ${\widetilde{\textbf{M}}}$ by $\widetilde{M}_0=1$ and $\widetilde{M}_k=k!\nu _k^k$ for $k\in \mathbb {N}$, in particular $\widetilde{M}_1\ge 1$. It follows that ${\widetilde{\textbf{M}}}$ satisfies (2.1) and (2.5). We need to point out, that we cannot conclude that ${\widetilde{\textbf{M}}}$ satisfies (1.1) and therefore cannot assume that ${\widetilde{\textbf{M}}}$ is a weigth sequence. But a close inspection of the proof of Theorem 2.14 in both the Roumieu and Beurling case shows that we still obtain the assertion of Theorem 2.14 for the sequence ${\widetilde{\textbf{M}}}$. But since ${\widetilde{\textbf{M}}}\approx \textbf{M}$, i.e. $\mathcal {E}^{[ \textbf{M} ]} ( \Omega )=\mathcal {E}^{[ {\widetilde{\textbf{M}}} ]} ( \Omega )$ and $\mathcal {E}^{[ \textbf{M} ]} ( \Omega ; P )=\mathcal {E}^{[ {\widetilde{\textbf{M}}} ]} ( \Omega ; P )$, we have in fact shown the assertion of Theorem 2.14 for $\textbf{M}$. Therefore the conjecture is also true for Denjoy–Carleman classes.

#### Remark 5.7

In view of the proof of Proposition 2.5 and the argument above, it would make sense to adapt the definition of a weight sequence by replacing (1.1) by the following condition:

$$\begin{aligned} \text {the sequence }\root k \of {M_k}\text { is increasing.} \end{aligned}$$

However, (1.1) is a standard assumption for weight sequences in context of Denjoy–Carleman classes, see e.g. [25] or [34] for classes given by weight matrices. Moreover, we have needed the concept of logarithmic convexity for the proof of Proposition 2.5.

## Footnotes

1 Here we understand by an ultradifferentiable structure an algebra of smooth functions defined by estimates on the derivatives and which contains the analytic class.

2 We use the notation $[*]=\{*\},(*)$, $*$ denoting a weight sequence, weight function or weight matrix, in order to write down statements in the Roumieu and Beurling case simultaneously.

3 The same is true of course for the statements in Theorem 1.3 and Theorem 1.6.

4 It is inspired by [36, Lemma 8].
